# Improving Adherence and Outcomes in Early Onset Schizophrenia: A Case Series on Role of Clozapine and Long-Acting Injectables for Refractory Courses

**DOI:** 10.7759/cureus.100996

**Published:** 2026-01-07

**Authors:** Mayank Gupta, Michael Esang, Angelica Antai, Faiza Zubiar, Priyal Khurana

**Affiliations:** 1 Psychiatry and Behavioral Sciences, Southwood Psychiatric Hospital, Pittsburgh , USA; 2 Psychiatry and Behavioral Sciences, Clarion Psychiatric Center, Clarion, USA; 3 Epidemiology, Mailman School of Public Health, Columbia University, New York, USA; 4 Psychiatry, The Trenton Psychiatric Hospital, Trenton, USA; 5 Psychology, Christ University, Ghaziabad, IND

**Keywords:** adolescents, antipsychotic medication, clozapine, early onset schizophrenia, long-acting injectables

## Abstract

Early onset schizophrenia (EOS) is a rare and severe psychiatric disorder associated with marked functional impairment and a complex diagnostic trajectory. Comorbid anxiety, mood dysregulation, and substance use further complicate clinical presentation and contribute to a more refractory course. Clozapine is widely acknowledged as the most efficacious treatment for individuals unresponsive to conventional antipsychotic therapy. However, its use in adolescents remains limited despite strong evidence of substantial clinical benefits, given the insidious and pernicious nature of this disorder. Although formal trials in adolescents are limited, observational data and clinical practice suggest that this combined approach may offer meaningful advantages for youth with persistent psychosis.

This case series describes six adolescents between 15 and 17 years of age who presented with severe psychotic symptoms, recurrent aggression, suicidality, or significant functional decline despite multiple adequate trials of oral antipsychotic medications. Clozapine was initiated in each case, and four patients also received a long-acting injectable antipsychotic (LAI) after establishing oral tolerability. Across cases, clozapine alone or in combination with an LAI was associated with substantial improvement in positive symptoms, behavioral regulation, and overall clinical stability, allowing transition to the least restrictive settings.

These findings illustrate the potential usefulness of clozapine and clozapine combined with LAI medications in adolescents with treatment-resistant EOS. They highlight the importance of early consideration of clozapine within a comprehensive treatment framework and point out the need for rigorous prospective research to guide clinical practice.

## Introduction

Early onset schizophrenia (EOS), defined as onset before age 18 years, and childhood-onset schizophrenia (COS), before age 13 years, are not categorized as distinct disorders in Diagnostic and Statistical Manual of Mental Disorders-5 (DSM-5) but instead represent points along a continuum of early neurodevelopmental vulnerability. Schizophrenia is rare in prepubertal children, with onset more typically emerging in mid-adolescence and following an insidious trajectory that often begins with affective or behavioral symptoms resistant to standard treatment [1,2]. The transition from early affective disturbances to frank psychosis is subtle, diagnostically challenging, and frequently delayed by the absence of objective biomarkers and family hesitancy toward accepting a severe psychiatric diagnosis.

Although debated, clinical observations and neurodevelopmental research suggest that earlier onset may be associated with a more severe course, marked by prominent structural and maturational anomalies in the brain [3-7]. Disruptions in cortical maturation and gray-matter development are consistently reported in early onset cases and are thought to underlie the cognitive impairment, emotional dysregulation, and poor functional outcomes commonly observed. 

This vulnerability is reflected in high rates of comorbid anxiety, mood dysregulation, cognitive deficits, and treatment resistance, all of which contribute to long-term disability. Diagnostic uncertainty, family distress, limited access to specialized services, and poor adherence further complicate early management. While second-generation antipsychotics remain the first-line treatment, a substantial subset of adolescents shows inadequate response [8-10]. Clozapine is the gold standard for treatment-resistant schizophrenia, yet its use in youth remains limited due to monitoring requirements, safety concerns related to brain development, and variable clinician comfort [2].

Growing clinical interest has focused on augmenting clozapine with long-acting injectable antipsychotics (LAIs) to improve adherence and reduce relapse risk. However, empirical evidence in adolescents is scarce. This case series contributes to the limited literature by presenting outcomes of six adolescents with severe, treatment-resistant schizophrenia treated with clozapine alone or in combination with LAIs.

## Materials and methods

This case series describes six adolescents (ages 15-17) diagnosed with EOS according to DSM-5 criteria and treated at a tertiary child and adolescent psychiatry service. The inclusion criteria were: (1) diagnosis of schizophrenia with onset before 18 years of age; (2) treatment resistance defined as inadequate response to at least two antipsychotics at appropriate doses and duration; and (3) initiation of clozapine with or without adjunctive LAIs. Patients with substance-induced psychosis, primary mood disorders, neurological conditions, or incomplete documentation were excluded.

Safety monitoring followed institutional protocols. Outcomes were assessed using documented clinical impressions, behavioral observations, hospitalization patterns, and functional changes.

## Results

Case 1

The initial case involves a 15-year-old Caucasian male patient admitted to our medical facility due to auditory and visual hallucinations, coupled with suicidal ideation. His referral for inpatient psychiatric treatment followed medical clearance from a local emergency department that included a complete blood count, complete metabolic profile, thyroid function test, Vitamin B12, folic test, urine toxicology, and urine analysis, which were all within normal range. Additionally, no neuroimaging studies were performed. Upon admission, the patient reported command auditory hallucinations instructing self-harm and visual perceptions of figures and insects. These symptoms had persisted for approximately one month, during which he contemplated suicide. He disclosed an incident of aggressive behavior toward his mother, prompted by auditory hallucinations, leading to the necessity for acute inpatient care. The patient, ordinarily non-violent, was not on any medications at the time of admission.

His psychiatric history revealed chronic feelings of being observed and followed, participation in a partial hospitalization program the previous year, a diagnosis of disruptive mood dysregulation disorder, and a prescription for fluoxetine. His past history revealed prodromal symptoms such as paranoia, irritability, and depression, but he had not experienced psychotic episodes until recently. The pertinent medical history encompassed pectus excavatum, cluster headaches or migraines, and a possible subliminal hyperthyroid function. A strong family history of mental health issues included diagnoses of bipolar disorder in his mother and sister, along with a paternal cousin's suicide. Born prematurely, the patient faced neonatal lung maturation challenges but achieved developmental milestones appropriately. Socially, he participated in online education and had been experiencing grief following his grandmother’s recent death. He also reported being sexually active. He denied any history of abuse, substance use, or alcohol consumption.

The mental status examination at admission depicted a disheveled Caucasian adolescent male patient with latent speech, marked psychomotor retardation, depressed mood, and a blunted affect. He exhibited circumstantiality, thought blocking, persecutory delusions, command auditory hallucinations, and suicidal thoughts, with impaired insight and judgment during crises. Subsequently diagnosed with schizophreniform disorder and comorbid generalized anxiety disorder, he was admitted to the inpatient child and adolescent unit. Treatment commenced with oral sertraline and olanzapine.

The patient exhibited a poor response despite adherence to the initial pharmacologic regimen of fluoxetine given 40 mg once daily, sertraline 100 mg once daily, and quetiapine started at 100 mg and increased to 400 mg. Self-harm precautions were implemented due to scratching and biting behaviors, and hallucinations were exacerbated. A second opinion was sought for treatment resistance. The treatment plan included risperidone, olanzapine, and chlorpromazine, which were tried separately at doses up to 6 mg, 25 mg, and 400 mg, respectively. The medications were cross-tapered, and Depakote levels were measured, showing a therapeutic level of 90.

The medical team also included divalproex sodium extended-release for mood stabilization. Therefore, they tapered olanzapine and initiated clozapine. Regular monitoring of complete blood count (CBC) with differential, medication titration for tolerability, and valproic acid levels ensued. The patient responded positively, leading to discharge with outpatient follow-ups, therapist and psychiatrist appointments, and a recommended MRI due to the first psychosis episode. The patient was discharged with his family, and the instructions stressed the importance of meticulous bloodwork monitoring for clozapine and sodium valproate. Medications included divalproex sodium extended-release 500 mg oral at bedtime, melatonin 9 mg oral at bedtime, Clozaril 125 mg oral at bedtime, and Risperdal 2 mg oral twice daily. The maximum dose of risperidone caused extrapyramidal effects, so it was reduced. Clozapine was used at lower doses with parental consent.

Case 2

The second case involves a 17-year-old Caucasian male patient in 11th grade, admitted to our hospital due to non-adherence with his LAI for several months. Profound confusion and thought disorder hindered information gathering, and his family reported episodes of wandering, getting lost, declining school attendance, and deteriorating academic performance. The patient did not have any delusions but was persistently paranoid and refused to engage in any medication trial in the past. The patient was tried on quetiapine and sertraline. Eventually, his inability to function in the community prompted hospitalization. The patient's history included a prior hospitalization, after which he had been prescribed Abilify Maintena once a month, after having been diagnosed with schizophrenia at age 15. While there was no significant family history, the patient regularly used cannabis and had recently experimented with lysergic acid diethylamide (LSD) before admission.

Upon admission, the patient was alert and oriented but perplexed about his hospitalization. He exhibited a flat affect, thought blocks, loose associations, and intense preoccupation with auditory hallucinations of the 2nd and 3rd person type, which included responding to stimuli. The patient reported that his schoolteachers were telling him to do homework, which made him feel petrified. Paranoid delusions concerning himself and his family were present, but he remained guarded about suicidal or homicidal tendencies. Memory and cognitive functioning appeared to be average; however, his insights into his illness and judgment were markedly compromised. A diagnosis of schizophrenia and cannabis use disorder was established, and the initial treatment plan involved reintroducing aripiprazole with a transition to aripiprazole lauroxil. The patient demonstrated non-adherence to aripiprazole monohydrate (Abilify Maintena) while in the community in the past three months prior to the admission and initially did not provide consent for long-acting injectable treatment. Subsequently, the patient agreed to initiate oral aripiprazole. Following oral stabilization, treatment was transitioned to long-acting aripiprazole lauroxil at a dose of 441 mg. However, the patient showed minimal clinical response to this formulation. During hospitalization, the patient remained floridly psychotic, combative, and violent due to paranoid delusions and visual/auditory hallucinations, especially those involving cats. As-needed antipsychotics included chlorpromazine (200 mg), quetiapine (up to 400 mg), and olanzapine (20 mg). He was prescribed aripiprazole monohydrate for a month but refused to take it after a few months, though he showed improvement, and this medicine was re-trialed after two years; therefore, we wanted to start with a low dose of oral medications. Risperidone was thus introduced (at 2 mg, going up to 4 mg), and upon establishing oral tolerability, a transition to paliperidone palmitate (Invega Sustenna) occurred. Persistent psychosis led to the addition of clozapine, titrated to 300 mg orally at bedtime, resulting in remission. The patient was then moved to a lower level of care, specifically a community residential rehabilitation program (CRR), where they would still get mental health care but in a less serious setting.

Case 3

This case involves a 17-year-old African American male patient who voluntarily presented to our facility, expressing concerns about psychotic symptoms. Referred by his outpatient physician due to four months of auditory and visual hallucinations, religion-themed delusions, and medication non-adherence, the patient's frequent violent outbursts and admitted impulses to harm others prompted this 7th inpatient psychiatric hospitalization. Diagnosed with schizophrenia, attention-deficit/hyperactivity disorder (ADHD), and cannabis use disorder, he had a family history of schizophrenia and a past of physical abuse from his father. Living with his mother and two brothers, he struggled academically, with declining grades.

Upon presentation, the patient exhibited guarded behavior, minimal interaction, response to internal stimuli, an irritable mood, and poor impulse control. He endorsed auditory hallucinations, religious preoccupation, and persecutory delusions. Poor insight and markedly impaired judgment led to a diagnosis of schizophrenia and cannabis use disorder, warranting admission to the child and adolescent inpatient psychiatric unit. The admitting psychiatrist obtained consent to commence clozapine, taking into account the patient's history, aggression, suicidal ideation, and inadequate response to prior antipsychotic treatments (olanzapine, risperidone, and paliperidone). Oxcarbazepine was added for mood stabilization.

Hospitalization saw a gradual clinical response, with an episode of epididymitis successfully treated with oral levofloxacin. As-needed chlorpromazine addressed aggression. Monitoring included absolute neutrophil count and clozapine levels. The patient engaged in the therapeutic milieu, exhibiting clinical progress, enabling transition to a lower level of care. Remission of psychotic symptoms and thoughts of lethality and improved insight characterized his recovery. Demonstrating appropriate attention span, psychomotor activity, organized thoughts, and congruent speech, he completed independent activities of daily living and participated positively in social interactions. He was discharged with a clozapine level of 312 ng/mL. His medications included Levaquin 500 mg orally once a day for five days for epididymitis, melatonin 9 mg orally at bedtime for insomnia, Trileptal 150 mg orally twice a day for mood stabilization, and Clozaril 275 mg orally at bedtime for psychosis. He was also scheduled to see his outpatient psychiatrist.

Case 4

A 17-year-old Caucasian male patient arrived at our facility under a commitment initiated by his legal guardians following a petition by his mother, resulting in his police-assisted transportation to the local emergency department. The primary concerns were auditory hallucinations, disorganized thinking, rambling speech, violent thoughts towards family, intermittent suicidal ideation without a clear plan, poor personal hygiene, reduced dietary intake leading to weight loss, and recent substance use, including peyote, LSD, and marijuana. He believed in possessing special powers and had run away from home, citing a unique connection to a friend. There was no history of psychiatric problems, abuse, or neglect, but the student was having trouble in school in the 11th grade. Family history only noted a non-contributory benign tumor in his father.

Upon admission, he presented with agitation, an irritable mood, labile affect, slow-latent speech, internal preoccupation, loose and disorganized thoughts, and various delusions. Suicidal and homicidal threats and impaired insight and judgment were observed. His calculated BMI was 19, leading to an initial diagnosis of an unspecified schizophrenia spectrum and other psychotic disorders. There was no neuroimaging done because of the patient’s initial refusal, but it was recommended for later, along with follow up with a neurologist and a psychiatrist. Substance-induced psychosis was not considered because the symptoms started much earlier than the use of substances, and the family reported that the patient used substances to self-medicate, but there was a clear demarcation between symptoms and absence of using substances. Admitted to the child and adolescent inpatient psychiatric unit, treatment commenced with oral paliperidone for psychotic symptoms and sodium valproate for mood stabilization.

Throughout the hospital stay, residual psychotic symptoms and episodes of aggression persisted. Paliperidone was titrated up to 12 mg with partial clinical response. At the family’s request for a second opinion, clozapine augmentation was initiated. Oral clozapine was initiated after obtaining a baseline absolute neutrophil count. The patient transitioned to paliperidone palmitate (234 mg, then 156 mg once a month), while continuing oral clozapine (200 mg), achieving remission of psychosis. Because of intolerance, oxcarbazepine was given instead of valproic acid, which had a level of 42 mmol/L. Discharge medication included melatonin for insomnia, Trileptal for mood stabilization, Clozaril and paliperidone palmitate for psychosis, and benzoyl peroxide for acne. A clozapine level of 259 ng/mL was recorded.

Upon discharge, the patient was symptom-free, non-aggressive, and engaged in prosocial behaviors. Medication and outpatient referrals, including psychiatric and primary care appointments, case management, and drug and alcohol services, were provided. The patient went back home to his family.

Case 5

A 16-year-old biracial male patient presented to our hospital under involuntary commitment due to command auditory hallucinations instructing him to take his life. It was noted that he did not adhere to the prescribed risperidone after a previous inpatient hospitalization, and no specific reason for this non-adherence was provided. He had falsely accused his mother of abuse and neglect, a realization that emerged by the time of admission. Experiencing guilt, depression, and suicidal thoughts, he intermittently smoked cannabis. Deteriorating school performance and a fatherless upbringing characterized his history.

Upon admission, he displayed orientation but appeared disheveled and malodorous, with blank stares and a guarded affect. Latent speech, constricted affect, disorganized thought process, paranoid delusions, and responses to internal stimuli were observed. Vague responses regarding suicidal thoughts were given, with intact memory and average intellect. The evaluation indicated diminished insight and judgment, gynecomastia, underweight status (BMI 18), and the presence of cannabinoids in urine toxicology. Diagnosed with schizophreniform disorder, treatment commenced with oral olanzapine (5 mg at bedtime) and oral Ensure for nutritional support.

Inpatient treatment showed limited response to olanzapine (10 mg twice daily), as-needed chlorpromazine, and sodium valproate for mood stabilization. The persistence of breakthrough agitation and psychotic symptoms led to a second opinion that recommended the addition of clozapine. Monitored for neutrophil count and valproic acid levels, clozapine was introduced, leading to remission of psychotic symptoms, reduced suicidal and homicidal thoughts, improved insight, and fewer auditory hallucinations. Stable for lower-level care transition, discharge included valproic acid (40 mcg/mL), clozapine (37 mcg/L), and the medications MiraLAX, Depakote ER, melatonin, Clozaril, and olanzapine.

At discharge, the patient was on Depakote ER 750 mg for mood stabilization, melatonin 10 mg for sleep, Clozaril 25 mg, and olanzapine 10 mg for psychosis. At the time of discharge, the patient received a prescription to taper olanzapine; however, the parents had a satisfactory outpatient follow-up, and cross-titration continued, resulting in the patient being on 200 mg of clozapine and 5 mg of olanzapine. The idea was to eventually taper off olanzapine. Follow-up appointments were scheduled at an outpatient program for early psychosis and with the primary care physician. The patient showed signs of clinical recovery and was considered stable when he was discharged.

Case 6

This case involves a 17-year-old male patient of Central Asian descent undergoing his second lifetime psychiatric hospitalization. He was referred after a suicide attempt via overdose on olanzapine and escitalopram tablets. Previously treated with aripiprazole and fluoxetine for depression, he remained profoundly depressed, expressing regret over surviving the attempt and contemplating further self-harm. He reported olfactory, tactile, and visual hallucinations, triggered by stressors like declining grades in virtual school. His father, with a history of mental health issues, recently obtained 50/50 custody. The patient admitted occasional cannabis use and possible past LSD use. In this case, the patient initially presented with symptoms consistent with attenuated psychosis syndrome (APS), characterized by attenuated psychotic features that overlapped significantly with depressive symptomatology, making early diagnostic clarification challenging. However, later on, clear evidence emerged that these symptoms were consistent with schizophrenia. 

Upon admission, he presented with psychomotor retardation, flat affect, and paranoid delusions, believing his father's phone was bugged. Diagnosed with a major depressive disorder with psychotic features and cannabis use disorder, he was stabilized in the child and adolescent inpatient psychiatric unit. Initial pharmacotherapy adjustments included increasing aripiprazole and fluoxetine doses.

Further evaluation identified schizophrenia criteria met, leading to a primary diagnosis change. Poor response to aripiprazole and risperidone prompted clozapine initiation, monitored with EKG, metabolic panel, and absolute neutrophil count (ANC). Despite prior aggression, clozapine gradually resolved symptoms, rendering him stable for lower-level care. Discharge included lorazepam for agitation, polyethylene glycol for constipation, and clozapine for psychosis. He was discharged with outpatient psychiatrist follow-up, resources for the clozapine clinic, and access to integrated behavioral health services. At discharge, he denied having suicidal thoughts, showed minimal psychotic symptoms, and demonstrated progress in his clinical recovery.

Table 1 summarizes the demographic and diagnostic characteristics of the six cases. Table 2 details presenting symptoms, treatment course, neuroimaging findings, and clinical outcomes.

**Table 1:** Demographic and clinical characteristics of the case series ADHD: Attention-deficit/hyperactivity disorder; LAI: Long-acting injectable antipsychotic

Variable	Case 1	Case 2	Case 3	Case 4	Case 5	Case 6
Age (years)	15	17	17	17	16	17
Sex	Male	Male	Male	Male	Male	Male
Race/Ethnicity	Caucasian	Caucasian	African American	Caucasian	Biracial	Central Asian
Primary Diagnosis	Schizophreniform disorder	Schizophrenia	Schizophrenia	Schizophrenia spectrum disorder	Schizophreniform disorder	Schizophrenia
Comorbidities	Anxiety disorder	Cannabis use disorder	ADHD; Cannabis use disorder	Substance use disorder	Cannabis use disorder	Major depressive disorder; Cannabis use disorder
Prior Antipsychotics	Olanzapine, risperidone, haloperidol, chlorpromazine	Aripiprazole (oral and LAI)	Olanzapine, risperidone, paliperidone	Paliperidone	Olanzapine, risperidone	Aripiprazole, risperidone
Clozapine Used	Yes	Yes	Yes	Yes	Yes	Yes
LAI Used	No	Yes (aripiprazole → paliperidone palmitate)	No	Yes (paliperidone palmitate)	No	No
Clinical Response	Improvement	Remission	Remission	Remission	Improvement	Improvement

**Table 2:** Clinical course, interventions, and outcomes across six adolescent patients SSRI: Selective serotonin reuptake inhibitor; LSD: Lysergic acid diethylamide

Case	Presenting Symptoms	Prior Hospitalizations	Substance Use	Treatment Course (Key Adjustments)	Clozapine Dose at Discharge	Neuroimaging	Outcome at Discharge
1	Command auditory hallucinations, visual hallucinations, suicidal ideation, aggression	Partial hospitalization (1 prior)	None	Initial SSRI + olanzapine → risperidone, haloperidol, chlorpromazine, divalproex sodium → clozapine initiation	125 mg qHS	MRI recommended (first-episode psychosis)	Marked reduction in hallucinations and suicidality; stable for outpatient care
2	Thought disorder, paranoia, wandering behavior, functional decline	≥1 prior inpatient admission	Cannabis; recent LSD use	Aripiprazole → aripiprazole lauroxil → risperidone → paliperidone palmitate → clozapine added	300 mg qHS	Not reported	Remission of psychosis; transferred to community residential rehabilitation
3	Auditory/visual hallucinations, religious delusions, aggression, homicidal ideation	≥6 prior hospitalizations	Cannabis	Failed olanzapine, risperidone, paliperidone → clozapine + oxcarbazepine	275 mg qHS (level 312 ng/mL)	Not reported	Resolution of psychosis and aggression; improved insight and functioning
4	Hallucinations, disorganization, delusions of special powers, aggression, substance-induced psychosis	None	Peyote, LSD, cannabis	Paliperidone + valproate → clozapine augmentation → paliperidone palmitate + clozapine	Clozapine level 259 ng/mL	Not reported	Full remission; non-aggressive; discharged home
5	Command auditory hallucinations, suicidality, paranoia, poor self-care	≥1 prior inpatient admission	Cannabis	Olanzapine + valproate → persistent symptoms → clozapine added	Clozapine level 37 mcg/L	Not reported	Resolution of suicidality and hallucinations; stable for outpatient care
6	Suicide attempt, psychotic depression, paranoia, multimodal hallucinations	1 prior hospitalization	Cannabis; possible LSD	Aripiprazole, risperidone (ineffective) → clozapine initiation	Not specified	EKG + metabolic monitoring	Significant symptom resolution; denied SI at discharge

## Discussion

EOS presents a complex, clinically challenging condition, predominantly affecting adolescents with a potentially severe course of illness. The prevalence of EOS sharply rises after age 14, particularly impacting males, highlighting a critical developmental period vulnerable to psychiatric disturbances. Neuroimaging studies underscore structural abnormalities in EOS, revealing cortical thinning and gray matter deficits in the prefrontal, insular, and temporal cortices. These regions play a fundamental role in higher-order thinking and emotional regulation [3-6]. Disruption in their maturation has been associated with treatment resistance and poor functional outcomes, directly contributing to the disorder's pathophysiology and underscoring the necessity of early intervention and specialized care and mitigating risks and long-term outcomes [7]. Diagnosing EOS remains complex due to symptom overlap with other psychiatric conditions and diagnostic uncertainties in the early stages. These factors contribute to delays in treatment initiation and highlight the importance of timely intervention strategies. Comorbidities such as anxiety disorders and substance use further complicate clinical management, necessitating comprehensive assessment and personalized treatment plans. Access to specialized neuroimaging and mental health services remains a critical issue, particularly in rural areas where disparities in healthcare provision persist, impacting timely diagnosis and intervention.

As pharmacotherapy is considered one of the early intervention strategies, second-generation antipsychotics are most preferred given the extrapyramidal symptoms that follow the first-generation antipsychotics [8-10]. Moreover, children and adolescents are at a higher risk of developing extrapyramidal symptoms such as sedation and weight gain [11,12]. These patients were in the hospital for a short period of time; the idea was that these patients would transition to a low level of care with the expectation that they can be switched to only one medication, but given the severe mental illness, the idea was to stabilize and then pursue the same treatment in outpatient care. 

Clozapine has widely been accepted as a gold standard for treating treatment-resistant schizophrenia [10]. Evidence supports that it improves positive and negative symptoms and aggression and additionally aids in reducing the risk of suicidal behavior. It has also been widely well tolerated, with manageable side effects in children and adolescents [13-15].

Adding LAIs to the regimen with clozapine may further improve its effectiveness. Table 1 summarizes current treatment strategies and effectiveness. The combination has been found to positively correlate with attaining remission and reducing relapses, the number of days of hospitalization, and emergency department visits [11,16,17]. A similar pattern has also been observed in these cases, where adding LAIs such as paliperidone with clozapine leads the way for remission of psychotic symptoms.

Regular monitoring of CBC has ensued during the inpatient treatment regimen [18]. The improvements followed a lower level of care with discharge and outpatient follow-ups. Clozapine and LAIs are used in adults and treatment-resistant conditions, but few presentations are in adolescents. The presented cases represent promising results by improving prognosis.

Effective management of EOS requires a multimodal approach that combines pharmacotherapy with psychosocial interventions. Cognitive behavioral therapy (CBT) is recommended as the primary intervention for individuals at elevated risk, along with second-generation antipsychotics tailored to their specific symptoms. In individuals who do not respond to two second-generation antipsychotics, clozapine is particularly effective for treatment-resistant cases. Integrating LAIs into early intervention services shows promise in reducing relapse rates and hospitalizations while also addressing ongoing challenges in the management of EOS that, often like this case series, is resistant to treatment as usual. 

## Conclusions

This case series underscores the persistent difficulties in diagnosing and managing EOS within conventional clinical practice. The gradual and diverse progression of symptoms, along with inadequate illness awareness and overlapping psychosocial stressors, perpetuates diagnostic ambiguity and leads to prolonged periods of untreated psychosis, a recognized indicator of adverse clinical and functional outcomes. Our findings underscore the importance of systematically distinguishing prodromal or attenuated psychotic states from fully syndromic EOS, and they emphasize the clinical relevance of early clozapine consideration in individuals exhibiting emerging resistance. The addition of LAIs for adolescents with persistent non-adherence resulted in quantifiable improvements in symptom stabilization and community functioning. Even in settings with limited resources, coordinated care components, especially structured family engagement, iterative consent procedures, and focused psychoeducation, were vital to sustaining treatment continuity and minimizing relapse risk.

Even though there have been many improvements in the nosology, neurodevelopmental understanding, and treatment paradigms of EOS, there are still big gaps in early identification, long-term results, and fixing systemic access problems in specialized care. Future research should focus on creating validated early detection models that combine clinical, cognitive, and biomarker-based risk factors; assess the efficacy and scalability of coordinated specialty care for younger demographics; and investigate the impact of digital phenotyping, passive monitoring, and AI-assisted decision-making tools in improving diagnostic accuracy and adherence support. Longitudinal studies are also necessary to enhance criteria for early clozapine initiation, identify predictors of response in adolescents, and ascertain optimal strategies for the integration of psychosocial interventions with pharmacological treatments throughout developmental stages. Advancing these research initiatives is crucial for enhancing the long-term prognosis, functional adaptation, and quality of life for individuals with EOS.
